# Extending the new era of genomic testing into pregnancy management: A proposed model for Australian prenatal services

**DOI:** 10.1111/ajo.13814

**Published:** 2024-04-05

**Authors:** Alice Rogers, Lucas De Jong, Wendy Waters, Lesley H. Rawlings, Keryn Simons, Song Gao, Julien Soubrier, Rosalie Kenyon, Ming Lin, Rob King, David M. Lawrence, Peter Muller, Shannon Leblanc, Lesley McGregor, Suzanne C. E. H. Sallevelt, Jan Liebelt, Tristan S. E. Hardy, Janice M. Fletcher, Hamish S. Scott, Abhi Kulkarni, Christopher P. Barnett, Karin S. Kassahn

**Affiliations:** ^1^ Paediatric and Reproductive Genetics Unit Women's and Children's Hospital Adelaide South Australia Australia; ^2^ Faculty of Health and Medical Sciences University of Adelaide Adelaide South Australia Australia; ^3^ Technology Advancement Unit Genetics and Molecular Pathology, SA Pathology Adelaide South Australia Australia; ^4^ Genetics and Molecular Pathology, SA Pathology Adelaide South Australia Australia; ^5^ Genomics Unit Genetics and Molecular Pathology, SA Pathology Adelaide South Australia Australia; ^6^ Pathology Queensland, Royal Brisbane and Women’s Hospital Brisbane Queensland Australia; ^7^ ACRF SA Cancer Genome Facility Genetics and Molecular Pathology, SA Pathology Adelaide South Australia Australia; ^8^ Maternal Fetal Medicine Service (MFMS) Women's and Children's Hospital Adelaide South Australia Australia; ^9^ Repromed Monash IVF Adelaide South Australia Australia

**Keywords:** congenital abnormalities, exome, genomics, prenatal diagnosis, rapid

## Abstract

**Background:**

Trio exome sequencing can be used to investigate congenital abnormalities identified on pregnancy ultrasound, but its use in an Australian context has not been assessed.

**Aims:**

Assess clinical outcomes and changes in management after expedited genomic testing in the prenatal period to guide the development of a model for widespread implementation.

**Materials and methods:**

Forty‐three prospective referrals for whole exome sequencing, including 40 trios (parents and pregnancy), two singletons and one duo were assessed in a tertiary hospital setting with access to a state‐wide pathology laboratory. Diagnostic yield, turn‐around time (TAT), gestational age at reporting, pregnancy outcome, change in management and future pregnancy status were assessed for each family.

**Results:**

A clinically significant genomic diagnosis was made in 15/43 pregnancies (35%), with an average TAT of 12 days. Gestational age at time of report ranged from 16 + 5 to 31 + 6 weeks (median 21 + 3 weeks). Molecular diagnoses included neuromuscular and skeletal disorders, RASopathies and a range of other rare Mendelian disorders. The majority of families actively used the results in pregnancy decision making as well as in management of future pregnancies.

**Conclusions:**

Rapid second trimester prenatal genomic testing can be successfully delivered to investigate structural abnormalities in pregnancy, providing crucial guidance for current and future pregnancy management. The time‐sensitive nature of this testing requires close laboratory and clinical collaboration to ensure appropriate referral and result communication. We found the establishment of a prenatal coordinator role and dedicated reporting team to be important facilitators. We propose this as a model for genomic testing in other prenatal services.

## INTRODUCTION

Fetal structural abnormalities can be readily detected on antenatal ultrasonography. In Australia, chromosomal microarray (CMA) is currently recommended as the first‐tier genetic investigation in the setting of fetal structural abnormalities.^1^ CMA improves the diagnostic yield of standard karyotype in this setting by approximately 6% by also detecting sub‐microscopic copy number variants (CNVs) in addition to abnormalities detectable by karyotyping.^2^ Diagnostic pick‐up rate is dependent on the presence, type and number of congenital abnormalities.^3^, ^4^

Over recent years, genomic testing, in particular whole exome sequencing (WES), has transformed diagnostic testing in the setting of suspected genetic disease in children and adults. In paediatric cohorts with suspected genetic disease, rates of diagnosis range between 26.5% for singleton (individual) tests, to 34.3% for trio tests (analysing parental genomic data, together with that of the individual).^5^ Similarly, genomic studies on late pregnancies, terminated pregnancies or stillbirths have reported diagnostic yields ranging from 10% to 50%, depending on the selected subgroup under investigation.^6^, ^7^, ^8^

Genomic testing was first utilised in the prenatal setting in 2012,^9^ although its clinical utility has historically been limited by cost and turn‐around‐time (TAT). WES, whereby only the ‘coding’ portion of the human genome is sequenced, has been particularly attractive. Two recent prospective cohort studies reported diagnostic rates of WES in pregnancies with structural abnormalities of 15.4% in fetuses affected by multi‐system anomalies and 3.2% in fetuses with isolated increased nuchal translucency.^10^, ^11^ The majority (61.5%) of causative variants identified arose *de novo*, ie spontaneously during gametogenesis without being inherited from either parent.^10^ More recently, a systematic review and meta‐analysis of 72 reports and 4350 fetuses demonstrated the benefit of pre‐selection of cases that were more likely monogenetic in aetiology by multi‐disciplinary review.^12^ It also allowed for the closer examination of the diagnostic yield of prenatal WES in a variety of phenotypic sub‐groups, with an overall incremental yield of WES being 31%, ranging from 53% in skeletal dysplasias to 2% for isolated increased nuchal translucency.^12^

Prenatal WES is logistically complex in that the samples (CVS or amniocentesis) often require cell culture before a sufficient sample is available for testing, and the need to be checked for maternal cell contamination, and WES is typically only considered after a negative microarray result. Adding to complexity, most clinical laboratories are set up to report WES results with a TAT of 6–12 weeks, appropriate for most paediatric testing. These factors combined have meant that results from prenatal WES are often not reported to families in the setting of an ongoing pregnancy but instead are provided for the purposes of reproductive planning, recurrence risk estimation and perinatal medical management.^10^, ^11^ There are very few reports of rapid prenatal WES and its impact on pregnancy management prior to 24 weeks gestation.^13^, ^14^, ^15^ To date, relatively few studies have assessed the impact of routine prenatal WES on clinical management or its applicability across health services in Australia,^13^, ^14^, ^15^, ^16^, ^17^ although there is a current Australian research project (PreGen) designed to address some of these issues.

South Australia (SA) has approximately 20 000 live births annually. The SA Pathology Cytogenetics Department performs prenatal microarray, karyotyping, and fluorescent *in situ* hybridisation aneuploidy testing. In July 2020, SA Pathology introduced rapid diagnostic WES testing in second trimester pregnancies. Result delivery was targeted to occur before 23 weeks gestation, because of local legal and ethical restrictions around pregnancy management beyond this gestation. Here, we report on the laboratory and clinical outcomes, including change in management, of this testing in 43 prospective families, as well as the logistics of setting up this service in our centre. These results contribute to our understanding of the benefits and risks of rapid prenatal genomic testing for pregnancy management prior to 23 weeks gestation.

## MATERIALS AND METHODS

### Referral pathway for prenatal genomic testing

Pregnancies considered to be at high risk for a genetic condition are referred to the Paediatric and Reproductive Genetics Unit (PRGU). Specific fetal presentations (eg skeletal dysplasia, non‐immune fetal hydrops) are offered targeted gene panels. Prenatal (trio) WES is offered where second trimester fetal anomalies are highly suspicious of a genetic aetiology and where a genomic diagnosis has the potential to impact clinical management. Families are eligible for rapid trio WES independent of gestational age. However, given the potential impact on clinical management, early referral is preferred.

The majority of potential cases are reviewed in a weekly multi‐disciplinary meeting, where opinions are sought from paediatric and obstetric subspecialists prior to referral to the PRGU. All referred cases are reviewed by senior clinical geneticist in close consultation with subspecialties, including maternal fetal medicine. Severe abnormalities, presence of multiple changes on ultrasound or bilateral symptoms are assessed against probability of an underlying genetic cause. Families who already opted for termination of pregnancy for other reasons are offered standard TAT WES. Tests were funded by the local hospital service with no cost to patients This study reviewed prenatal exome referrals in our service between July 2020 and July 2022.

### Establishment of a rapid prenatal exome service

The impetus to provide a rapid prenatal exome service for women/couples grappling with the possibility of a genetic condition was the firm upper limit of 22 + 6 weeks gestation for termination of pregnancy in SA. Furthermore, as pregnancy advances, decision making becomes psychologically and logistically more complex.

**Figure 1:**
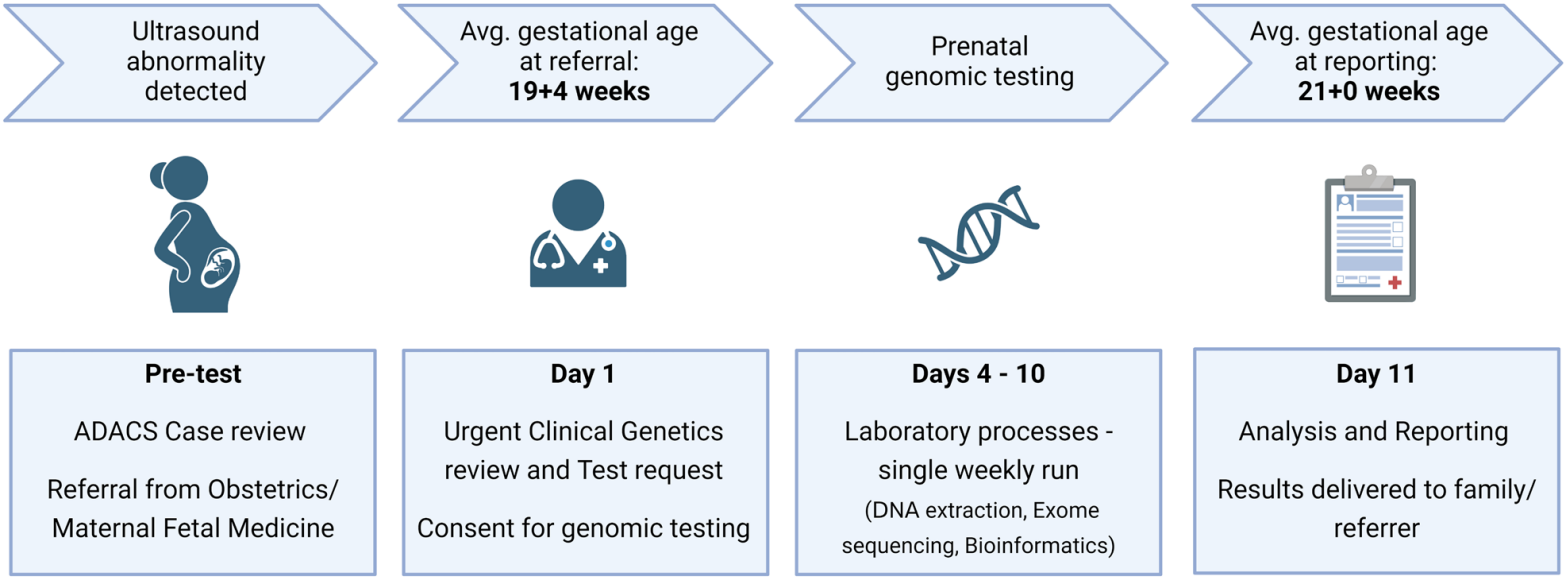
Prenatal genomic testing pathway.

SA Pathology was already accredited for exome testing and experienced with rapid genomic testing to assist in neonatal and paediatric intensive care consultations.^18^ These clinical and laboratory workflows were reviewed and applied to the prenatal setting to allow for rapid turn‐around of results and reporting prior to 23 weeks gestation, wherever possible (Fig. 1). A senior medical scientist was assigned the role of prenatal coordinator to coordinate sample collection and delivery to the cytogenetic and molecular laboratories and communicate with the clinical services. A dedicated analysis and reporting team was also set up.

The choice of CVS (*n* = 10) or amniocentesis (*n* = 33) samples and the requirement for a completed microarray was dependent on the timing of the antenatal presentation. Maternal cell contamination studies were performed using CMA data for amniocentesis and the AmpFlSTR Identifiler kit (Thermofisher Scientific) for CVS samples. This enabled the exclusion of maternal cells introduced during sample collection, or amplified via cell culture, which could interfere with fetal genotyping.

### Genomic testing and clinical reporting

Genomic testing was performed using SA Pathology standard clinically accredited WES protocol which incorporates a sample identity check and parentage confirmation. This method detects single nucleotide variants and small insertion/deletion variants within exons, including splice site regions. Variant annotation was performed using SA Pathology's in‐house clinical interpretation support tool Variant Grid v3. Variant pathogenicity was based on American College of Medical Genetics reporting guidelines.^19^ For prenatal testing, generally only pathogenic or likely pathogenic variants related to fetal phenotype are reported. Clinical consultation was sought upon the identification of highly suspicious variants of uncertain significance (VUSs), which was relevant in two cases (see Results). Analysis was performed on variants that satisfied expected inheritance patterns in all published Mendelian disease genes (approx. 6650 genes). Incidental findings are discussed during pretest counselling and are only reported following clinical review and discussion with the referring clinician.

### Audit of clinical outcomes during pregnancy and after birth

Ethics approval was obtained via the Women's and Children's Hospital Human Ethics Committee (1257A/05/2025) to perform an audit of the clinical outcomes during pregnancy and after birth by retrospectively accessing medical records held at the PRGU. Families with a clinically significant molecular finding were separately approached and consented for participation in this study with emphasis on change in management data. Impact on management was determined by assessing pregnancy outcome (termination vs continuation) and subsequent pregnancies. Other clinical outcome measures included ultrasound findings, termination/birth details, neonatal phenotype (including autopsy findings, where applicable) and hospital length of stay.

## RESULTS

Rapid prenatal genomic testing was performed on 43 prenatal samples comprising 40 trios, two singletons and one duo analysis. One sample failed testing due to poor DNA quality and one sample was analysed post‐termination of pregnancy. Of the 41 successful tests performed and reported during an ongoing pregnancy, diagnostic findings were reported in 15 fetuses (37%). Fourteen out of 15 were classified as pathogenic or likely pathogenic. The clinical interpretation of VUSs became important in two fetuses. In one fetus, compound heterozygous variants were identified in *RAPSN* (one likely pathogenic and one VUS), in which the VUS was ultimately reported after consultation with the requesting geneticist. In another fetus, compound heterozygous VUSs in *EXOSC3* were also reported after consultation with the requesting geneticist showed compelling clinical correlation to phenotype (Table 1). Of these 15 findings, eight were confirmed *de novo*, five compound heterozygous for an autosomal recessive disorder, one homozygous recessive and one X‐linked recessive. Prenatal genomic testing was requested concurrently with the microarray in 55% of cases.

**Table 1:** Diagnostic findings from rapid prenatal genomic testing and pregnancy outcomes. Other genomic diagnoses not included in the clinical outcome audit, include *DNAL1* (Primary ciliary dyskinesia 16, MIM: 614017), *SPAST* (Spastic paraplegia 4, MIM:182601) and *FGFR3* (Thanatophoric dysplasia, MIM:187600). MIM numbers refer to the Online Mendelian Inheritance in Man disease entries (https://www.omim.org/)

ID	Genomic diagnosis	Morphology scan findings	TAT (days)	Gestation at reporting (weeks)	Gene and inheritance	Pregnancy outcome (gestational age)	Recurrence risk	Future pregnancy; future prenatal testing
8	Pontocerebellar hypoplasia (MIM 614678)	CH, DWM, small CBM, AVSD, FEB, hypoechoic liver, MGN, RGN	15	20 + 5	*EXOSC3* (VUS; VUS) Inherited; compound heterozygous; AR	TOP (22 + 0)	High (1:4)	Y (ongoing pregnancy); No
10	Nemaline myopathy 2 (MIM: 256030) Spinocerebellar ataxia 47 (MIM: 617931)	TEV, LB <2centile, ↓EFW, Cavum vergae	13	21 + 4	*NEB* (P) Inherited; compound heterozygous; AR *PUM1*(P) *De novo*; heterozygous; AD	TOP (21 + 3)	High (1:4) Low (1%)	Y (delivered); PND (*NEB* heterozygous)
15	Lymphatic malformation 6 (MIM: 616843)	HF, SCO, PE	11	21 + 6	*PIEZO1* (LP; LP) Inherited; compound heterozygous; AR	TOP (22 + 6)	High (1:4)	N IVF; considering PGT‐M
18	Skraban‐Deardorff syndrome (MIM:617616)	NB, ↓EFW	8	18 + 2	*WDR26* (P) *De novo*; heterozygous; AD	TOP (19 + 1)	Low (1%)	N
24	Campomelic dysplasia (MIM:114290)	↑NF, LL MSM, TEV, ↓FL (<1centile), ABN thorax (↑RL, ↑APø)	13	21 + 5	*SOX9* (P) *De novo*; heterozygous; AD	TOP (22 + 1)	Low (1%)	Y (delivered); N
25	Smith‐Lemli‐Opitz syndrome (MIM:270400)	BL PAP, ↓ EFW	12	22 + 0	*DHCR7* (P;P) Inherited; compound heterozygous; AR	TOP (22 + 1)	High (1:4)	Y (spontaneous pregnancy prior IVF); PND (also affected; SAP <12/40)
30	Sotos syndrome 1 (MIM:117550)	UNL VM, ↑CSF, ↑ NF, BL RPD	10	22 + 0	*NSD1* (LP) *De novo*; heterozygous; AD	TOP (22 + 5)	Low (1%)	N
34	Fetal akinesia deformation sequence 2 (MIM 618388)	MGN, HF, IUGR, AMC, PLH	11	26 + 4	*RAPSN* (P; VUS) Inherited; compound heterozygous; AR	Delivered, died 1 h life (37 + 0)	High (1:4)	N
36	Leri‐Weill dyschondrosteosis (MIM:127300) and X‐linked chondrodysplasia punctata (MIM: 302950)	RZM LB, MFH	15	22 + 4	*SHOX* (P)*, ARSL* (P) Maternally inherited; XLR	Delivered and surviving (38 + 0)	High (1:2 for males)	N
37	Noonan syndrome (TA/TB) (MIM 163950)	DCTA triplets (TA/TB MCDA) TA:CH, PE, ABN IVC, PLH, BP, HF, LGA. TB:BP, DJS. TC:NAD	13	20 + 0	*PTPN11* (P) *De novo*; heterozygous; AD	Delivered; TA/TB died, day 82 life; TC surviving (29 + 0)	Low (1%)	N
38	Noonan syndrome (MIM 163950)	DJS, PE	16	18 + 2	*PTPN11* (P) *De novo*; heterozygous; AD	TOP (19 + 1)	Low (1%)	Y; <12/40; considering PND

Abbreviations: ⌀, diameter; ↑, increased; ↓, decreased; ↑, CSF, prominent cerebrospinal fluid spaces; ABN, abnormal; AD, autosomal dominant; AMC, arthrogryposis multiplex congenital; AP, anterior–posterior; AR, autosomal recessive; AVSD, atrioventricular septal defect; BL, bilateral; BP, bulky placenta; CBM, cerebellum; CH, cystic hygroma; DCTA, dichorionic triamniotic; Del, delivered; DJS, dilated jugular sacs; DWM, Dandy‐Walker malformation; EFW, estimated fetal weight; FEB, fetal echogenic bowel; FL, foot length; GxPx, gravida and parity; HF, hydrops fetalis; IUGR, intrauterine growth restriction; IVC, inferior vena cava; IVF, *in vitro* fertilisation; LB, long bones; LL, lower limb; LP, likely pathogenic; MCDA, monochorionic diamniotic; MFH, mid‐face hypoplasia; MGN, micrognathia; MSM, mesomelia; N/A, not available; NAD, no abnormality detected; NB, absent nasal bone; NF, nuchal fold; NT, nuchal translucency; P, pathogenic; PAP, postaxial polydactyly; PE, pleural effusion; PGT‐M, pre‐implantation genetic testing for monogenic disorders; PLH, polyhydramnios; PND, prenatal diagnosis; RGN, retrognathia; RL, rib length; RPD, renal pelvis dilatation; SAB, spontaneous abortion (<12/40); SCO, subcutaneous oedema; TA, twin/triplet A; TAT, turn‐around‐time; TB, twin/triplet B; TC, triplet C; TEV, talipes equinovarus; TOP, termination of pregnancy; UNL, unilateral; US, ultrasound; VM, ventriculomegaly; VUS, variant of uncertain significance; XLR, X‐linked recessive.

### Real‐time reporting prior to 23 weeks

Samples were referred for rapid prenatal testing at a median gestational age of 19 + 5 weeks (range 15–25 weeks) The median TAT for reporting of prenatal trio exome was 12 days (range 7–21 days), resulting in a median gestational age at time of reporting of 21 + 3 weeks, with the latest case being reported at 26 + 4 weeks gestation. This fetus was compound heterozygous for variants in the *RAPSN* gene, and treatment options were appraised for management of the ongoing pregnancy and birth.

### Clinical outcomes study

**Figure 2:**
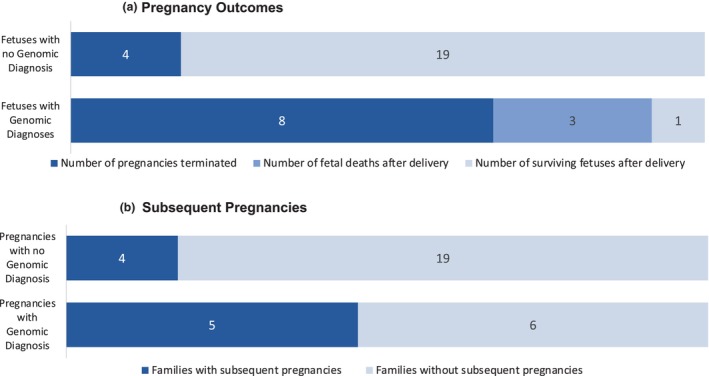
(a) Pregnancy outcomes (ie, number of terminations, numbers of deaths after delivery and the number of newborns surviving after delivery) for families who did or did not receive a diagnosis from rapid prenatal genomic testing, as measured by the number of terminations, deaths after delivery and the number of babies surviving after delivery. (b) Number of subsequent pregnancies reported in families who did or did not receive a diagnosis from rapid prenatal genomic testing as of October 2022.

Of the 43 cases in which genomic testing was performed, 34 families were included in the audit of clinical outcomes; the remaining families either did not provide consent (two), were uncontactable (one), did not have accessible medical records (three) or had to be excluded for other reasons (three). Of the 34 families included in the clinical outcome study, 11 families (12 fetuses) received a diagnosis while 23 families had no genomic finding. Of the 11 families who received a diagnosis from early pregnancy genomic testing, eight (67%) opted for termination of pregnancy, three (25%) continued the pregnancy but the newborn did not survive, and one (8%) continued the pregnancy and the newborn was alive at eight months of age (Fig. 2a). In contrast, of the 23 families with no diagnosis from early pregnancy genomic testing, only four (17%) opted for termination of pregnancy on other grounds. Nineteen (83%) continued the pregnancy with subsequent neonatal survival.

At the time of audit, five of 11 families (45%) who had received a diagnostic finding were pregnant again. Four families (36%) were actively considering *in vitro* fertilisation (IVF) and pre‐implantation genomic testing for monogenic disorders (PGT‐M). In contrast, of the 23 families with no diagnosis, only four (17%) were pregnant again (Fig. 2b).

### Instructive case

Family 25 was referred at 20 + 1 weeks gestation following a morphology scan showing isolated bilateral polydactyly, and mildly reduced fetal growth. A non‐invasive prenatal test (NIPT) was low risk. The family had two other healthy children. The mother had late onset type 1 diabetes which was well controlled. The family were offered trio WES.

Prenatal exome testing identified compound heterozygous, pathogenic variants in *DHCR7*, diagnostic of Smith‐Lemli‐Opitz (SLO) syndrome (MIM: 270400). SLO is characterised by growth restriction, microcephaly, intellectual disability and multiple congenital abnormalities including cleft palate, cardiac defects, hypospadias in males, postaxial polydactyly and 2–3 syndactyly of the toes.^20^

Both parents are carriers for this autosomal recessive condition. The recurrence risk for this family in future pregnancies is thus one in four (25%). Upon learning of the genomic findings, the family opted for termination of pregnancy at 22 + 1 weeks gestation and declined autopsy. In the year after this, the family were referred for PGT‐M but fell pregnant prior to undergoing IVF. Based on the genomic diagnosis in the prior pregnancy, testing for SLO at 12 weeks of gestation could be offered in the subsequent pregnancy.

## DISCUSSION

Here we report on rapid and early prenatal genomic testing and clinical outcomes in 43 SA families referred during the second trimester. We discuss the feasibility and clinical utility of this service as a model for other Australian services.

WES established 15 diagnoses, spanning a broad range of fetal presentations, including skeletal abnormalities, fetal hydrops, multiple congenital abnormalities, and cardiac findings. All diagnoses were in genes previously associated with fetal anomalies and present in established gene panel lists. In two different families, VUSs in the *EXOSC3* and *RAPSN* genes were reported as the clinical phenotypes associated with the gene were specific and consistent in each respective fetus. Typically, VUSs are not reported by our service in the prenatal setting as these cannot be used for immediate clinical management and there is insufficient time for follow‐up studies to assist in evaluation of such variants. Given that 8/15 diagnoses were *de novo* and 6/15 diagnoses compound heterozygous requiring allelic phase for diagnosis, trio analysis of the fetus and both parents was essential in interpretation of these variants.

The results of our clinical data collection suggest that outcomes of WES resulted in change in management. The majority of families with a positive finding (67%) used the information from genomic testing to help inform termination of pregnancy decisions. Several families opted for pre‐implantation genetic testing or prenatal testing in future pregnancies to manage recurrence risk, demonstrating the clinical utility of having a molecular diagnosis. In contrast, families in whom no clinically significant genomic finding was made generally opted to continue the pregnancy (83%), suggesting that they were reassured by the lack of a clinically significant genomic diagnosis.

Also of note was that ongoing pregnancies with a molecular diagnosis had an increased mortality rate, when compared to those without. In the group of ongoing pregnancies with a clinically significant genomic diagnosis, three out of four resulted in death. In contrast, no deaths were reported in the families who continued the pregnancy after a non‐diagnostic genomic test result (19 families).

Reporting of rapid exome testing in the prenatal setting prior to 23 weeks gestation is logistically challenging. Structural ultrasound is typically performed at 20 weeks gestation to allow better discrimination of fetal features. With a standard microarray workflow taking 7–14 days and WES 7–14 days, this puts significant pressure on all teams involved to rapidly return results. Importantly, we were able to implement this rapid prenatal service without additional instrumentation or performing out‐of‐session sequencing runs; instead, we used the standard weekly laboratory workflow already part of our core diagnostic service. However, we found the establishment of a prenatal coordinator role highly effective in coordinating the clinical and laboratory teams and ensuring samples are received in the laboratory as soon as possible. The assignment of a dedicated analysis and reporting team was also important. To reduce TAT and depending on clinical context, microarray and rapid exome testing were often concurrently ordered (55% of cases). This approach seems sensible in that the diagnostic yield of microarrays is generally much lower than that of genomic testing, unless for specific clinical presentations, such as SHOX (short stature homeobox‐containing gene) deficiency disorders which are typically only investigated by microarray. However, in one family in this study, microarray and genomic testing were performed in parallel due to advanced gestational age and the desire to rapidly exclude all possible causes of the fetal presentation, which included significant mid‐face hypoplasia in this fetus, resulting in both tests returning the result of a contiguous deletion involving the SHOX and ARSL (arylsulfatase L) genes. As sequencing costs decrease further, the referral pattern for microarray and genomic testing may change in the future. This may include the adoption of whole‐genome sequencing in the prenatal context, which would at the same time detect many CNVs currently detected by CMA.

Psychosocial aspects and family experience were not investigated in this study but are the focus of other ongoing research studies, including the PreGen project, in which our centre also participates. Nevertheless, the results from our clinical audit demonstrate that many families actively used the results from genomic testing in current and future pregnancy decision making. We have demonstrated that it is feasible to routinely return results prior to 23 weeks gestation, without the need for significant additional resources. We hope that our findings may be useful to inform the design of similar services in other centres across Australia, thus helping establish genomic testing more prominently for pregnancy management.

## Supporting information


**Table S1.** Detailed clinical and laboratory data for all pregnancies.
